# Spinning and Tactile Hand/Wear Comfort Characteristics of PET/Co-PET Hollow Fabrics Made of Inorganic Particles Embedded Sheath/3-Core Bicomponent Yarns

**DOI:** 10.3390/ma18225188

**Published:** 2025-11-14

**Authors:** Jiman Kang, Hyunah Kim

**Affiliations:** 1 Korea Textile Development Institute, Daegu 41842, Republic of Korea; jmkang@textile.or.kr; 2 Department of Clothing & Textiles, Changwon National University, Changwon-si 51140, Republic of Korea

**Keywords:** bicomponent yarns, inorganic particles, hollowness ratio

## Abstract

This paper reports the spinning and wear comfort properties of polyethylene terephthalate (PET)/copolymer-PET (Co-PET) hollow yarns and their fabrics, as well as the effect of the wt.% of inorganic particles embedded in the core of the bicomponent yarns. The results are discussed in terms of the types and amounts of inorganic particles (titanium dioxide (TiO_2_) and calcium carbonate (CaCO_3_)) embedded in the sheath of the bi-component yarns (Kolon semi-dull (KSD), Kolon full-dull (KFD), and Kolon calcium carbonate (KCC) PET/Co-PET yarns). The three sheath/3-core bicomponent yarns developed in this study exhibited good spinnability and weavability with relatively strong tenacity and breaking strain. Their optimal spinning conditions were determined. The KCC PET/Co-PET fabric showed the greatest hollowness ratio, followed by the KFD PET/Co-PET and KSD PET/Co-PET fabrics. This might be attributed to the higher wt.% (2.5 wt.%) of CaCO_3_ particles embedded in the sheath of the KCC PET/Co-PET yarns and to the larger particle size (0.8 μm) of CaCO_3_. Regarding the wear comfort, the moisture management system (MMT) test indicated that the KFD PET/Co-PET fabric is suitable for market applications because of its good moisture absorption and rapid drying. The KFD PET/Co-PET fabric is useful for winter clothing applications because of its relatively high heat retention rate and lack of durability issues with washing. An examination of the wearing performance for fitness with a tactile hand feel showed that KFD and KCC/Co-PET fabrics imparted a softer tactile hand feel than the KSD PET/Co-PET fabric. On the other hand, the KCC PET/Co-PET fabric was assumed to have some issues with wearing durability.

## 1. Introduction

Fiber spinning methods are generally classified into three categories: dry spinning, wet spinning, and melt spinning [1,2]. Of these, melt spinning is a widely used, sustainable, and cost-effective method for producing artificial fibers and filament yarns from thermoplastic polymers compared to dry and wet spinning. Bicomponent fibers with various structural configurations are generally produced using a combination of two different polymers. Many studies [3,4,5,6,7,8,9,10,11,12,13,14,15,16,17,18] have focused on fiber spinning technologies for producing bicomponent fibers. Spinning methods of the bicomponent fibers are divided as side-by-side [7,8,9], island-in-the-sea [12,15], segmented-pie [10,11,14,17], and core/sheath [5,6,16,18]. On the other hand, many review articles [19,20,21,22,23] have been published about natural polymer-based (Lyocell et al.), bio-based, and biodegradable polymers. Polylactic acid (PLA), polybutylene succinate (PBS), and polycaprolactone (PCL) are typically considered biodegradable thermoplastics that are produced on an industrial scale. Nevertheless, their poor physical properties limit their commercial applications. Although various laboratory-scale investigations have examined hollow bi-component fibers based on natural and bio-derived polymers, translating these findings into practical or commercially viable applications remains limited. Various hollow fibers developed as new functional fibers exhibit unique physical properties, including thermoregulation, filtration, and good water absorption, while remaining lightweight. Accordingly, hollow fibers have been used in high-performance applications, such as artificial dialysis membranes, water purification, and sportswear textiles. In particular, the hollow filaments produced by a conjugated melt-spinning method are used widely as sportswear materials because of their superior water absorption, thermal insulation properties, and light weight. Hollow filaments used in sportswear are mostly made from PET and nylon, and they are usually 20–25% lighter than conventional ones [5]. On the other hand, various hollow filaments are produced using bi-component yarn manufacturing technology on a conjugated melt spinning machine. Regarding the bicomponent fiber, the following were defined elsewhere [6]. The American Society of Testing Materials (ASTM) defines a bicomponent fiber as a fiber composed of two polymers that are chemically or physically distinct (or both) [3]. Yarn cross-sections available in bi-component yarns are divided into four types produced widely by textile companies: sheath/core, side-by-side, segmented pie, and sea-and-island. Of these cross-sections, the sheath/core type is the most commercially popular in sportswear, and PET/polyethylene (PE), PET/polybutylene terephthalate (PBT), PET/Co-PET, PET/polypropylene (PP), and PET/nylon types are currently commercialized.

In sportswear, however, superior water absorption and rapid drying combined with lightness are required, which can be achieved with high-hollow fibers. Moreover, their improved thermoregulation property makes them more comfortable to wear. Therefore, many Japanese companies, such as Unitica Textiles Ltd., Toray Industries, and KB Seiren Co. Ltd., have commercialized famous brands for high-hollowness fibers. Wincall^®^ is a sheath/core type nylon/PET bicomponent yarn produced by Unitica Textiles Ltd. The sheath and core parts of Wincall^®^ are composed of nylon and easily soluble PET (Co-PET), respectively, which are eluted by sodium hydroxide (NaOH) during the dyeing and finishing processes. On the other hand, two important processes after spinning the hollow fiber are yarn texturing to impart crimp to the yarns and elution treatment to achieve hollow sheath/core yarns. Of these, elution is achieved by alkali reduction with NaOH, resulting in good water absorption, rapid drying, and lightness. The elution process is essential for determining the tactile hand feel of fabrics because it varies with the elution conditions. In addition, texturing is required to impart a crimp to the yarns, thereby affecting the tactile hand of the fabrics. The spinning of high-hollow fibers to enhance wear performance and lightness while wearing clothing is closely related to texturing technology because the fiber surface is damaged during yarn texturing, making it challenging to develop high-hollow fibers that are also light. Accordingly, the spinning conditions of bi-component sheath/core yarns must be considered with yarn texturing and elution processes to produce high-quality hollow fibers.

Many studies [17,18,24,25,26,27,28,29,30] have examined sheath/core-type hollow fibers and their elution behaviors. Hu et al. [17] examined the characteristics of bicomponent anti-static fibers with sheath/core and segmented-pie configurations produced using PBT and PET mixed with a carbon black/dispersing agent. El-Salmawy et al. [24] examined the alkali treatment of PBT/PBSL core/effect fiber. In addition, El-Salmawy and Kimura [25] examined the alkali treatment of the PET/polybutylene succinate L-lactate (PBSL) and PET/poly L-lactic acid (PLLA) core/sheath filaments. They reported that their developed filaments can be applied to biomedical and marine fields. Nevertheless, previous studies did not examine the elution of the bicomponent yarn to obtain hollow fibers.

Some studies [28,29,30,31,32] on manufacturing nylon hollow fabrics from PET/nylon bicomponent yarns have been performed using caustic alkali reduction technology. Of these, Kim et al. [28,29] examined the physical properties of nylon hollow fabrics made from sheath/core type PET/nylon filament according to the elution condition during alkali reduction in the finishing process. Several studies [30,31,32] examined the weight reduction and dyeing characteristics of ultra-fine nylon fabrics made from sea/island type PET/nylon filaments based on the alkali reduction ratio (%) of the fabrics. On the other hand, many studies [33,34,35,36,37,38,39] have been carried out on the tactile hand and wear comfort of PET hollow fibers and their fabrics. Micropores (craters) formed on the fiber surface during the production of hollow yarns were formed using inorganic particles, such as TiO_2_ [27,28,29,30,31,32,33,34,35,36,37,38,39] and CaCO_3_ [40,41,42,43]. In particular, TiO_2_ has been used to produce PET and nylon hollow yarns. A review of the literature indicated that recent research trends exhibit four major deficiencies. Regarding the application of CaCO_3_ to modify PET and nylon fibers, the technique has been used primarily in the plastic industry, but it is still in its early stages in textile applications. No studies have examined the melt spinning of three-core (3-core) PET hollow fibers incorporating inorganic particles such as calcium carbonate (CaCO_3_), specifically for functional applications in sportswear. In addition, no research has been conducted on highly hollow, lightweight 3-core PET hollow fibers with a hollowness ratio of approximately 30% that simultaneously exhibit excellent moisture absorption, rapid drying capability, and enhanced heat retention. Moreover, the studies that have been published focused on the relationship between the processing conditions and the resulting fiber characteristics, with limited investigations into the high performance (superior moisture absorption and drying properties) of the high hollow fabrics (hollowness: 30%) applicable to the sportswear market. Despite these studies, few have examined sheath/3-core PET spinning and its elution technology because of confidentiality and patent issues. Moreover, few studies have examined the overall characteristics of spinning sheath/3-core PET bicomponent yarns in relation to the wear comfort properties of their fabrics. Accordingly, the present study aims to address these research gaps by systematically exploring the spinning behavior, physical performance, and overall material characteristics of CaCO_3_-embedded 3-core PET hollow fibers suitable for sportswear applications. Therefore, this study examined the spinning characteristics of the sheath/3-core PET/Co-PET bicomponent yarns with varying wt.% of inorganic particles (TiO_2_ and CaCO_3_) embedded in the core of the bicomponent yarns. Furthermore, the wear comfort characteristics, such as moisture absorption/drying and heat retention rate of the woven fabrics made from three types of sheath/3-core yarns with varying wt.% of inorganic particles, were measured and compared with the types and wt.% of the inorganic particles embedded in the yarn sheaths. In addition, the elution characteristics of the sheath/3-core PET/Co-PET yarn fabrics were examined to achieve a high hollowness PET yarn based on the elution conditions. Finally, the tactile hand feel of the hollow PET fabric was estimated with the wearing performance for fit while wearing clothing, based on the mechanical properties of the fabric.

## 2. Materials and Methods

### 2.1. Spinning of PET Bicomponent Yarns

**Figure 1:**
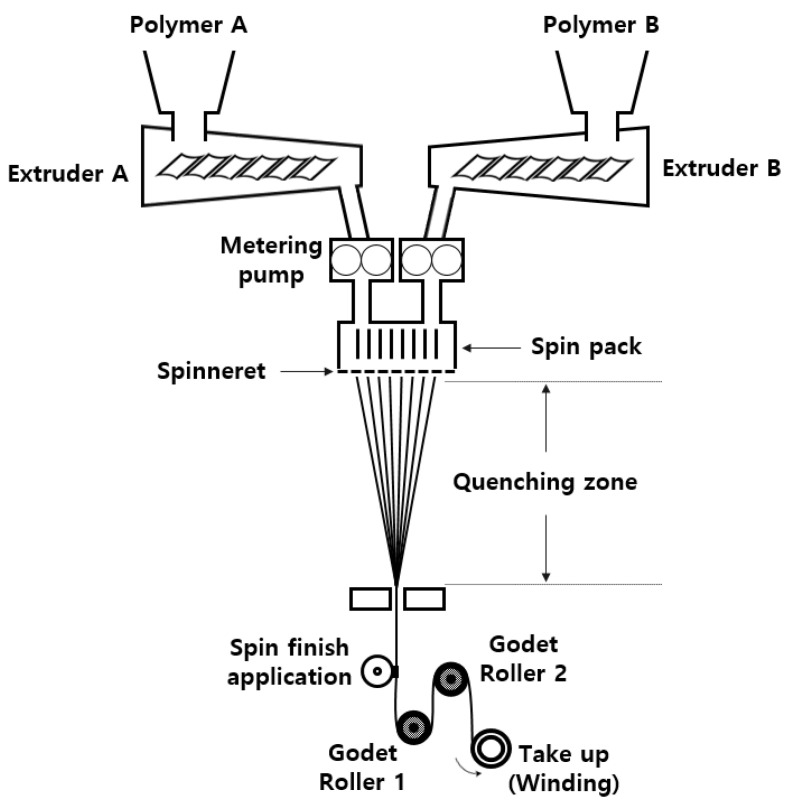
Schematic diagram of the conjugated spinning machine.

**Figure 2:**
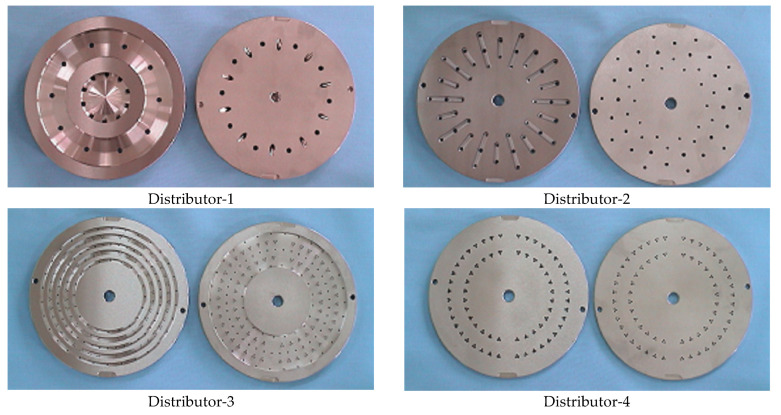
Newly designed distributors attached to the spin pack.

Three types of sheath/3-core type PET bicomponent spin draw yarns (SDY, 75d/34f) were spun on the conjugated spinning machine (TW-536, TMT Co., Ltd., Osaka, Japan). Figure 1 presents a schematic diagram of the conjugated spinning machine used in this study. An alkali-soluble PET (Co-PET) polymer in the core and PET polymer in the sheath were melted and mixed using a screw extruder from the feed hopper and then pumped and flowed to distributors (1, 2, 3, and 4) in the spin pack, where the sheath and core polymers are divided. Finally, the sheath/3-core PET bicomponent yarns were spun from a spinneret. Four distributors attached to the spin pack were newly designed in this study, as shown in Figure 2. The optimal parameter of the spinning nozzle is the L/D ratio (L = nozzle hole length, D = nozzle hole diameter). The flow rate of the processed polymer increases at a small L/D ratio, which may result in uneven fiber surfaces or die swell. The pressure loss also increases as the L/D ratio increases, and thermal degradation of the polymer may occur.

Yarn specimen 1 was composed of a PET Kolon semi-dull (KSD) polymer (70 wt.%) in the sheath and Co-PET polymer (30 wt.%) in the core. TiO_2_ (3600 ppm, 0.36 wt.%) was embedded in the PET KSD polymer in the sheath. Yarn specimen 2 was composed of the TiO_2_ (25,000 ppm, 2.5 wt.%) embedded PET Kolon full-dull (KFD) polymer in the sheath (70 wt.%) and Co-PET polymer in the core (30 wt.%). TiO_2_, 0.1–0.3 μm in size, was embedded in molten PET polymer, which helps produce craters (pores) more easily in the sheath of the yarns during elution. Yarn specimen 3 was made from CaCO_3_-embedded PET Kolon calcium carbonate (KCC) polymer in the sheath (70 wt.%) and Co-PET polymer in the core (30 wt.%). CaCO_3_ (25,000 ppm, 2.5 wt.%), 0.8 μm in size, was embedded in melted PET polymer in the sheath. Table 1 lists the bicomponent yarn specimens.

The melt viscosity in this study was measured using a capillary rheometer (Shimadzu Capillograph 2, Shimadzu Corp., Kyoto, Japan). The melt viscosity in the core ranged from 200 to 400 Pa·s, and that in the sheath ranged from 300 to 600 Pa·s. The optimal spinning temperature was determined as the temperature at which fiber formation was stable, no end-breakage occurred, and spinnability was satisfactory during the spinning process.

The spinning temperature in the spin pack of the yarn specimen 1 was 293 °C, and the spinning speed was 3910 m/min. The spinning temperature of yarn specimen 2 was 290 °C with a spinning speed of 3890 m/min and 290 °C with 3880 m/min for yarn specimen 3. The spinning temperature was adjusted according to the pack temperature. A spinneret was installed in the pack, where pressure adjustment and gel filtering in the polymer were performed during melt spinning. The filtration mesh was a 400-mesh stainless steel screen. Pressure fluctuations occurred upon the addition of the inorganic additive (TiO_2_). The initial spinning pressure ranged from 980 to 1200 psi, and the final pressure reached 2200 to 3000 psi. The mesh was typically replaced when the pressure exceeded 4000 psi. In this experiment, no mesh replacement due to clogging occurred because a pilot-scale spinning machine was used to produce a small amount of fiber. Table 2 lists the characteristics of the PET chips used in the sheath and core. The characteristics of the polymer used in this study are as follows. The intrinsic viscosities of the KSD, KFD, and KCC PET polymers in the sheath were 0.65, 0.63, and 0.655, respectively, and the viscosity of the Co-PET polymer in the core was 0.665. The glass transition temperatures of them were 80.7, 72.1, and 77.3 °C, respectively, and that of Co-PET was 81.3 °C. The melting temperatures of the KSD, KFD, and KCC PET polymers in the sheath were 252, 255.2, and 256.2 °C, respectively, and that of Co-PET was 248 °C.

### 2.2. Texturing of Bicomponent Yarns

Three types of spin draw yarns (SDY, 75d/34f) in Table 1 were textured to make draw-textured yarn (DTY, 75d/34f) under the following conditions on a belt-type texturing machine (33H, Murata, Japan): draw ratio of 1.05 by the surface velocity ratio between first and second feed roller; velocity ratio of 1.4 by the ratio between the feed speed and belt surface velocity; heater temperature of 170 °C and feed speed of 400 m/min.

### 2.3. Fabrication of Fabric Specimens

Considering the weavability of DTY 75d/34f, which was spun as sheath/3-core bi-component yarns (Table 1). Three types of warp yarns were interlaced with 75d/34f PET DTY and 30d/12f PET SDY to eliminate yarn breakage during weaving, and the resulting PET 105d/46f was used as warp yarns. For weft yarns, sheath/3-core bi-component yarns, 75d/34f, were folded, and three types of weft yarns (150d/68f) were also made. In addition, warp beams were made on a single warping machine (ROM2, Karl Mayer, Obertshausen, Germany) using interlaced 105d/46f bicomponent warp yarns.

Finally, fabric specimens were woven with three different warp beams using weft yarn specimens, 150d/68f sheath/3-core bicomponent yarns on a prototype rapier loom (Sambo Machinery Co., Ltd., Seoul, Republic of Korea). The weave pattern was plain. Table 3 lists the specifications of the woven fabric specimens.

### 2.4. Elution Experiment for Complete Elution

The optimal elution conditions for complete elution of the woven fabric specimens were decided after the preliminary elution experiment. The elution experiment was performed with varying temperatures and times under a fixed NaOH concentration. The NaOH concentration was 10 wt.%, and the elution temperature changed from 90 to 110 °C with 10 °C increments. The elution time was also changed from 0 to 60 min in 10 min intervals at each elution temperature. The optimal elution conditions for complete elution were a treating time of 50 min at 100 °C temperature in a 10 wt.% NaOH aqueous solution. The elution experiment used infrared (IR) dyeing equipment (DL-6000, Daelim Starlet, Siheung-si, Republic of Korea). The woven fabric specimens (20 × 20 cm) were cut and laid in the IR dyeing equipment, where they were eluted under the elution conditions listed in Table 4. The eluted fabrics were washed and dried at 40 °C in a heat dryer. The elution rate of the complete eluted fabric was calculated using Equation (1). Ten readings were carried out for each specimen.

Elution rate (%) = (A − B)/A × 100,(1)

where A is the mass of the fabric specimen before elution treatment, and B is the washed and dried mass of the fabric specimen after elution treatment.

### 2.5. Measurement of Yarn Physical Properties

The yarn linear densities of the three yarn specimens were measured using KSK ISO 2060 [44], and 20 tests were performed on each yarn. The tenacity and breaking strain of PET/Co-PET SDY and its DTY were measured using an Instron (Testometric Micro 350, London, UK). The unevenness (U%) of the three yarn specimens was measured using a Uster evenness tester (Tester 5, Uster Co., Uster, Switzerland). Wet thermal shrinkage was assessed after a 30-min heat treatment at 100 °C in a water bath and calculated from the lengths before and after the wet heat treatment. Scanning electron microscopy (SEM) was performed to observe a cross-section of the PET/Co-PET bi-component yarns and their fabrics. The alkaline hydrolyzed hollow PET yarns were also examined by field emission SEM (FE-SEM, S-4100, Hitachi Co., Ltd., Tokyo, Japan). Optical microscopy (i-Camscope 305A, Seoul, Republic of Korea) images of a fiber cross-section after spinning were taken using cut fibers on the conjugated spinning machine to detect the configuration of cross-section formation of the sheath/3-core fiber.

### 2.6. Measurement of the Hollowness Ratio of the Complete Eluted Fabric Specimens

The hollowness ratio is a critical property that affects the wear comfort, such as moisture absorption and rapid drying, as well as water vapor permeability and fabric lightness. The hollowness ratios of the three types of sheath/3-core fabric specimens with different sheath types (KSD, KFD, and KCC PET) were measured as follows. First, three types of sheath/3-core fabrics were treated in an aqueous 10 wt.% NaOH solution for 50 min at 98 °C. Hollow filaments were collected from these treated fabrics. Cross-sectional samples were prepared using a microtome to produce thin sections of the yarn cross-section, ensuring no loss of porosity. The hollow ratio of these thin sections was measured using an optical microscope and Image-Pro Plus^®^ v7.1 software (Media Cybernetics, Inc., Rockville, MD, USA). The hollowness ratio was calculated using Equation (2) from the area ratio between the hollow area (H) and the total cross-sectional area (T) of the yarns:

Hollowness (%) = H/T × 100.(2)

The areas of H and T were calculated from the diameters of the core and sheath, respectively, which were measured using microscopy and Image-Pro Plus^®^ v7.1 (Media Cybernetics, Inc., USA) program software. Sixty-eight readings per filament were repeated for ten filaments to calculate the average value.

### 2.7. Measurement of Moisture Absorption and Drying Properties

The moisture absorption and drying properties of the three types of hollow woven fabrics were measured using a moisture management tester (MMT). The measurements were taken using the standard AATCC (American Association of Textile Chemists and Colorists) Test Method 15 (AATCC, 2002) [45]. A detailed measuring method is reported elsewhere, and followings are cited from the prior study [39]. “Five fabric specimens were cut into 80 × 80 cm squares for use in the experiment. A special solution (0.15 g), mixed with distilled water and sodium chloride, was injected onto the top surface of each specimen.

**Figure 3:**
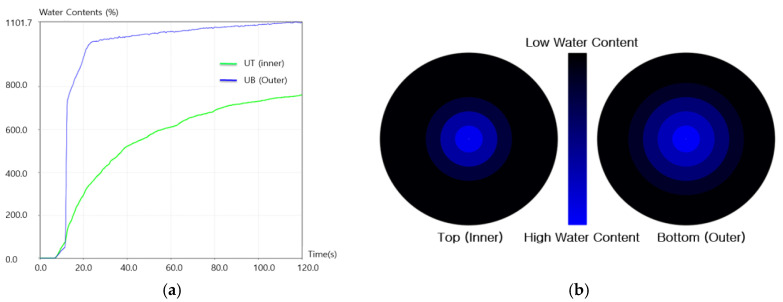
Diagram of the MMT experimental result: (**a**) moisture content vs. time and. (**b**) moisture location vs. time [46].

The electrical contact resistance of the fabric specimens changes when water is transported through a fabric. The wetting time(s) was measured on the top and bottom surfaces when the fabric first became wet. The absorption rate is defined as the average moisture absorption ability, which is calculated automatically from the initial slope of the water content versus the time curve shown in Figure 3a. In the MMT test, the absorption and diffusion of liquid droplets (test solution) through the upper and lower electrodes of the specimen are detected by changes in electrical resistance. The “Water Content (%)” value of the y-axis in Figure 3a represents the relative change in electrical conductivity compared to the reference, rather than the absolute amount of water. Therefore, this value is not the physical water content based on mass, but a standardized relative index of conductivity variation over time. The maximum wetted radius (MWR, mm) is the radius of the circle shown in Figure 3b, which is defined as the distance between the center of the wetted ring and the circle. The spreading speed (mm/sec) is defined as the velocity from the center of the wetted ring to the MWR, which is automatically calculated from MWR/t, where t is the time to reach the maximum wetted ring”.

### 2.8. Measurement of the Heat Retention Rate (I)

**Figure 4:**
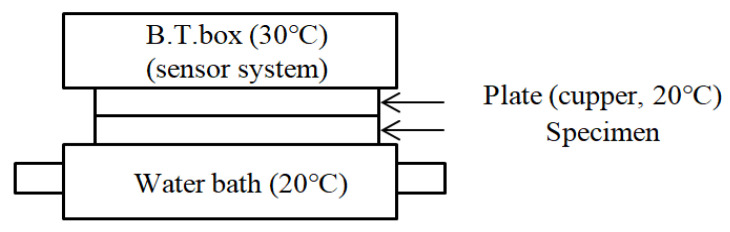
Schematic diagram of the measuring apparatus: KES-F7 [47].

Understanding how the hollowness of the sheath/3-core bicomponent yarns influences the heat retention rate of the fabric is critical to examining the thermal wear comfort of the PET/Co-PET hollow fabrics. The heat retention rate was measured using a KES-F7 (Kato Tech Co., Kyoto, Japan). Figure 4 presents a schematic diagram of the Kawabata Evaluation system (KES-F7 system) reported elsewhere [47]. First, the temperature in the B.T. box was set to 30 °C, and water at 20 °C was circulated in a water bath, where a fabric specimen had been placed. The heat retention rate (I) was assessed and calculated using Equation (3):

(3) $$I=\left(1-\frac{b}{a}\right)\times \;100,$$

where a is the heat in Watts (W) emitted from the plate shown in Figure 4, and b is the heat (W) emitted from the fabric specimen.

### 2.9. Measurement of Fabric Mechanical Properties

The tactile hand feel of the hollow PET fabrics is essential for sportswear applications, and the mechanical properties of the three types of hollow PET fabrics with different hollowness were measured using a Fabric Assurance Simple Testing (FAST) apparatus [48]. The surface thickness (ST, mm), as a measure of compressibility, was measured using the FAST-1 system, defined as the difference in fabric thickness at compressions of 1.96 cN/cm^2^ and 98.04 cN/cm^2^. The bending rigidity (B, μN·m) was measured and calculated from C obtained with a FAST 2 according to Equation (4):

B = W × C3 × 9.52 × 10^−6^, (4)

where C is the bending length (mm), and W is the weight per unit area of fabric (cN/m^2^). The extensibility (E, %) was measured at a load of 98.04 cN/cm using a FAST-3 system. The shear rigidity (G, N/m) was measured using a FAST-3 system and was calculated from EB 5 obtained by a FAST-3 using Equation (5).

G = (123/EB5) × 1(N/m)(5)

where EB5 is the bias extension under a 4.85 cN/cm width in %.

## 3. Results and Discussion

### 3.1. Physical Properties of PET/Co-PET Bi-Component Yarns (SDY and DTY)

The physical properties of yarns are essential for estimating the spinnability and weavability. The mechanical properties of yarn, such as tenacity and breaking strain, were measured and analyzed, along with the unevenness and wet thermal shrinkage of the yarn specimens, to predict weaveability and process conditions in the finishing process.

**Figure 5:**
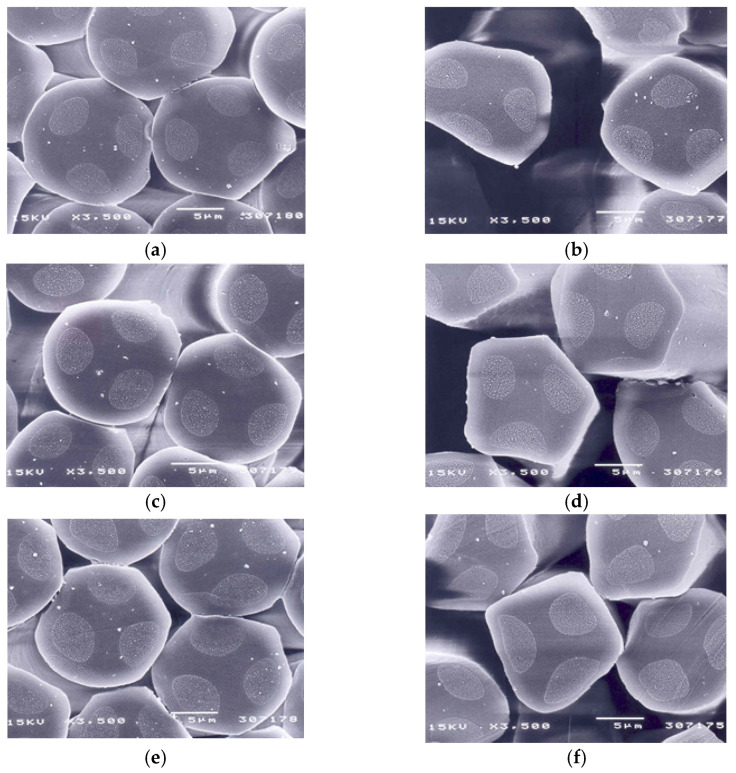
SEM images of the cross-section of three yarn specimens: (**a**,**b**): yarn specimen 1, (**c**,**d**): yarn specimen 2, (**e**,**f**): yarn specimen 3.

Table 5 lists the physical properties of three types of yarn specimens. The linear densities of the DTY were slightly higher than those of the SDY, as shown in Table 5. This was attributed to yarn crimp inserted into the DTY after texturing, resulting in a higher DTY linear density. The tenacity and breaking strain of the DTY yarn specimens were lower than those of the SDY, i.e., they were reduced after texturing, which was caused by damage to the yarn surface by the distortion of the constituent filaments caused by friction between yarns and the texturing device during yarn texturing, resulting in a decrease in the tenacity and breaking strain of DTY. Figure 5 presents SEM images of yarn cross-sections of three types of yarn specimens. Three cores in the sheath/3-core bicomponent yarns are shown. In addition, the circular cross-sections of SDY (Figure 5a,c,e) were changed to non-circular ones (Figure 5b,d,f) by the distortion caused by resistance to the frictional force between the yarn surface and the belt of the texturing machine. This distortion and some damage (crack and fission) of the yarn surface were assumed to cause a decrease in tenacity and breaking strain of the DTY, as described elsewhere [5]. In addition, the wet thermal shrinkages of DTY in Table 5 were 6–7% higher than those of the SDY, which was attributed to the thermal instability of DTY, i.e., DTY has internal thermal stress in the yarns induced by heat treatment received in the texturing process (which is unstable), resulting in high thermal shrinkage in the boiling water.

On the other hand, regarding the yarn physical properties according to the types of yarn specimens, the linear density of yarn specimen 1 (KSD PET/Co-PET) was lower than those of yarn specimens 2 (KFD PET/Co-PET) and 3 (KCC PET/Co-PET) (Table 5). This was attributed to the more embedded TiO_2_ particles (2.5 wt.%) in the yarns and greater particle size (0.8 μm) of the CaCO_3_ in the yarn specimen 3, resulting in a higher yarn linear density of yarn specimens 2 and 3 than 1, and yarn specimen 3 than 2. The tenacity and breaking strain of yarn specimen 1 were greater than those of yarn specimens 2 and 3, which was attributed to the lower unevenness of yarn specimen 1 than yarn specimens 2 and 3. The unevenness of yarn specimens 2 and 3 was higher than that of yarn specimen 1. The higher unevenness of yarn specimens 2 and 3 was assumed to be due to the greater inclusion (2.5 wt.%) of TiO_2_ particles and larger particle sizes (0.8 μm) of the CaCO_3_ particles embedded in the yarn specimens 2 and 3, respectively. Hence, the greater inclusion of particles and larger particle size in the polymer make extruded yarns much more uneven, resulting in a decrease in the tenacity and breaking strain of yarns. The physical properties of the bicomponent yarn specimens are essential for estimating the spinnability and weaveability in spinning and weaving processes. Therefore, the yarn tenacity and breaking strain of the three types of PET/Co-PET bicomponent yarns produced in this study are used to predict the spinnability and weavability. The tenacities of the three types of bi-component yarns ranged from 3.86 to 3.95 gf/d (Table 5), similar to that of the nylon/PET sheath/core bicomponent yarns [5]. The breaking strains were 28.8, 32.1, and 32.7% in SDY and 26.4, 28.4, and 30.8% in DTY produced in this study, similar to a previous study [5]. Hence, the spinnability and weavability of the three yarn specimens produced in this study are relatively good, i.e., there is no problem (no broken filament and no end breakage) in the spinning and weaving processes. Moreover, the tenacity and breaking strain of the three types of bi-component yarns appeared to be similar to the commercialized regular PET yarns [49], meaning there is no problem with the weaving process. Therefore, the current yarn production process is advantageous and applicable to the production of commercial textile goods. In addition, the wet thermal shrinkage values of the three yarn specimens (SDY) ranged from 8.4% to 10.7%, which were slightly higher than those of the regular commercialized PET yarns [49]. This was attributed to the bicomponent structure of the sheath/3-core PET yarns, resulting in increased wet thermal shrinkage because of the different interfacial surface energies between the sheath and core, which will attempt to increase the compressive and relaxed strains caused by wet heat [50]. The wet thermal shrinkage is critical for controlling the wet finishing process. Accordingly, this must be considered to decide the optimal process conditions for dyeing and finishing.

### 3.2. Elution and Hollowness Ratios of Three Hollow Fabric Specimens

**Figure 6:**
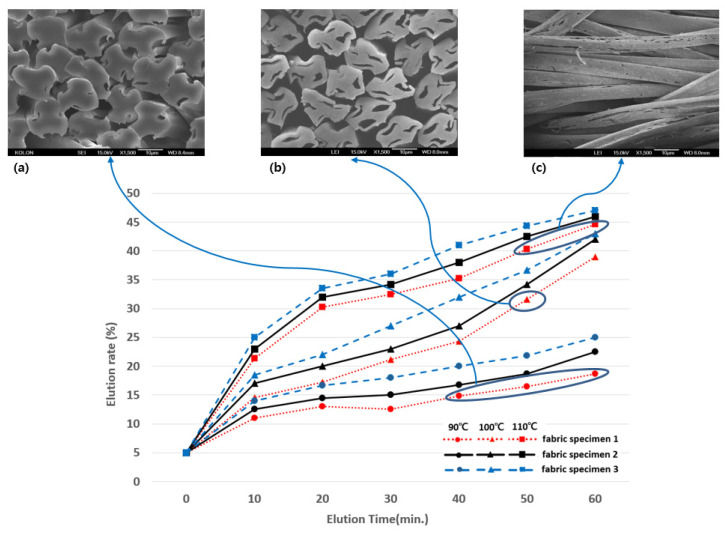
Elution rates of the fabric specimens according to the elution temperatures and time: (**a**) 80 °C, (**b**) 100 °C, (**c**) 110 °C.

A preliminary experiment was performed to determine the optimal elution rate and conditions for obtaining complete elution and high hollowness of the sheath/3-core bicomponent yarns. Figure 6 shows the elution rates of the fabric specimen 1 according to the elution temperatures (90, 100, and 110 °C) and time (0 to 60 min). The elution rate increased as the elution time increased, and complete elution was observed after 50 min of treatment at 100 °C and above 30 min at 110 °C. A similar trend was observed in fabric specimens 2 and 3. Figure 6b presents SEM images of the yarn cross-section of the complete elution treated for 50 min at 100 °C. The treatment temperature of 90 °C did not result in complete elution regardless of the treatment time, as shown in Figure 6a, and a broken wall on the surface of the yarns was observed after 50 and 60 min of treatment at 110 °C, as shown in Figure 6c. Similar broken walls on the yarn surface were also observed in fabric specimens 2 and 3, indicating incomplete elution defects rather than defects that occurred during elution. Accordingly, overall, the optimal elution conditions were a treatment time of 50 min at 100 °C in an aqueous 10 wt.% NaOH solution.

Table 6 lists the hollowness and elution rates of the three hollow fabric specimens treated at 100 °C and 50 min. Table 7 presents the analysis of variance (ANOVA) for each specimen, with average diameters (core and sheath) and hollowness within the 95% confidence interval. The average values between the fabric specimens were statistically significant, respectively, as F_0_ (V/Ve) > F(2, 27, 0.95) and *p* < 0.05. The hollowness ratios of the three hollow fabric specimens treated for 50 min at 100 °C were 25.8%, 27.1%, and 30.1%, respectively, and the elution rates were 31.6%, 34.2%, and 36.7%, respectively, as shown in Table 6. In particular, the hollowness ratio of specimen 3 (KCC PET/Co-PET) was 30.1%, similar to the hollowness ratio of the well-known Sumlon hollow fiber made by Toray in Japan [26]. Hollowness is important for achieving superior moisture absorption when wearing lightweight sportswear. This result suggests that the bicomponent hollow yarns fabricated in this study were successfully spun to predict and ensure excellent wear comfort properties. The hollowness ratios of fabric specimens 2 and 3 were greater than that of fabric specimen 1, which was attributed to the distortion on the yarn surface and cracks of cores in the yarns of fabric specimens 2 and 3 because of the high wt.% of TiO_2_ and CaCO_3_ particles embedded in the sheath of the sheath/3-core yarns. The distortion (or contraction) and cracks widened the core, and the overall fiber area was narrower, increasing the hollowness rate of fabric specimens 2 and 3.

**Figure 7:**
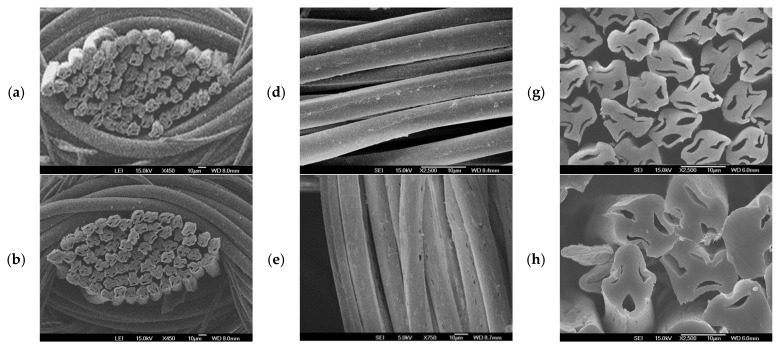

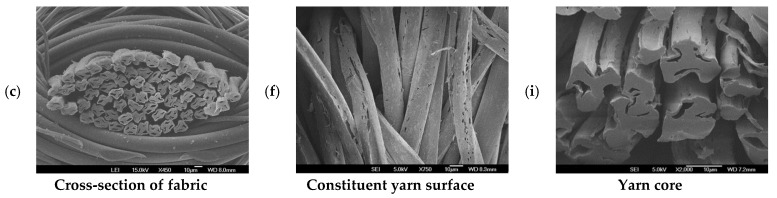
Distortion of the yarn surface and cracks of the core according to elution conditions: (**a**,**d**,**g**): specimen 1, (**b**,**e**,**h**): specimen 2, (**c**,**f**,**i**): specimen 3.

**Figure 8:**
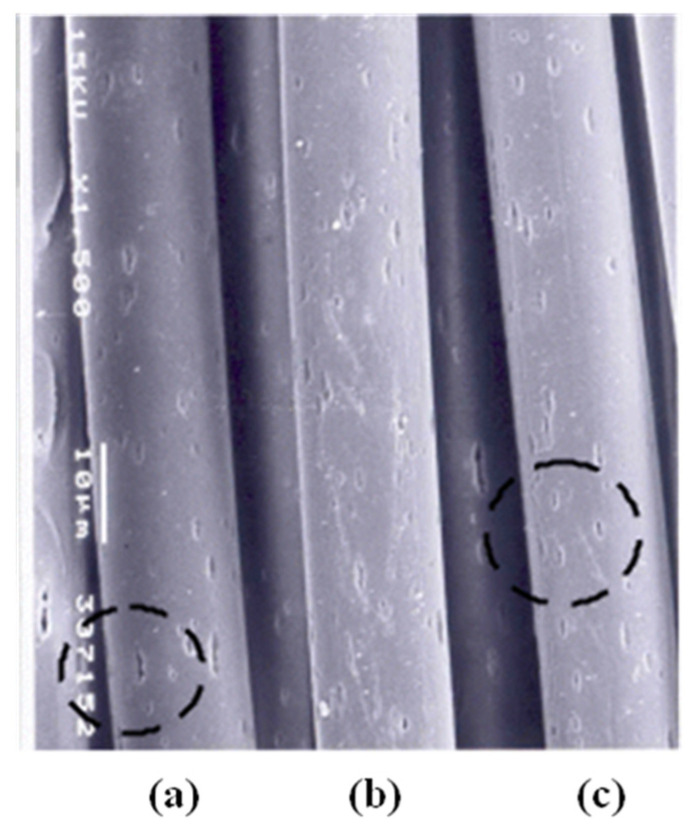
Side photograph of elution-type hollow filaments: (**a**) yarn specimen 1, (**b**) yarn specimen 2, and (**c**) yarn specimen 3.

Figure 7 presents the fabric cross-sections ((a),(b),(c)), cracks ((d),(e),(f)) of the yarn surface, and cracks of the core in the yarns of fabric specimens 1, 2, and 3. In addition, high wt.% of TiO_2_ and CaCO_3_ embedded in the sheath made the elution rates of fabric specimens 2 and 3 greater than that of fabric specimens 1. This was attributed to the many craters formed on the fiber surface because of the higher wt.% of TiO_2_ and CaCO_3_ embedded in yarn specimens 2 and 3 than in yarn specimen 1, which produced many eluted fragments and broken pieces from many craters on the fiber surface, resulting in higher elution rates of fabric specimens 2 and 3. This was verified by SEM images of the constituent fiber surface in each yarn of the three fabric specimens. Figure 8 presents SEM images of craters in the three eluted yarns (KSD PET, KFD PET, and KCC PET) of the three fabric specimens. As shown in Figure 8 (as indicated by the circled areas), more numerous and larger craters were observed on the yarn surface of KFD PET (specimen 2) and KCC PET (specimen 3) than those of KSD PET (specimen 1), resulting in a greater elution rate of fabric specimens 2 and 3.

Furthermore, the elution rate of fabric specimen 3 was greater than that of fabric specimen 2 because of the larger particle size (0.8 μm) of CaCO_3_ than that (0.1–0.3 μm) of TiO_2_, which made larger craters on the fiber surface and many eluted fragments and broken pieces after elution, resulting in a higher elution rate of fabric specimen 3.

### 3.3. Moisture Absorption and Drying Properties of the Three Fabric Specimens

**Figure 9:**
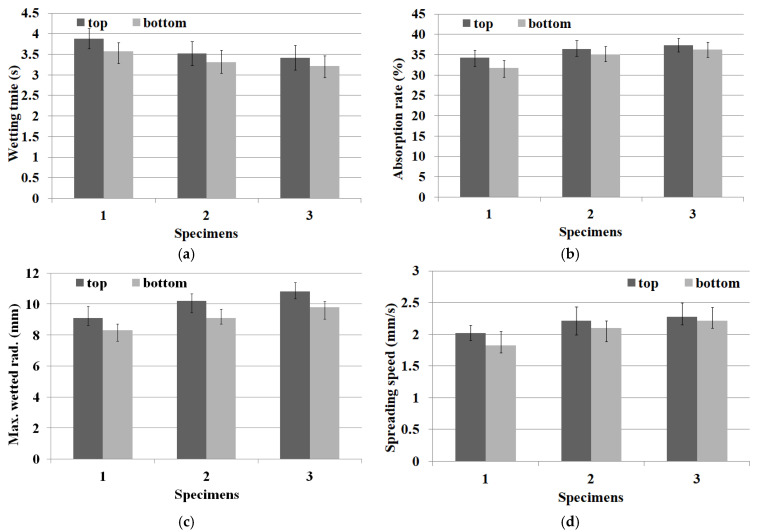
Diagram of MMT results of the fabric specimens: (**a**) wetting time, (**b**) absorption rate, (**c**) max. wetted rad, and (**d**) spreading speed.

Many studies have been conducted on the moisture absorption and drying properties of high-performance fabrics [46,51,52,53,54]. Of these studies, the MMT method was newly applied to evaluate the moisture transport behavior in multiple directions, including moisture absorption and drying properties [46]. Table 8 lists the moisture transport behaviors of the three fabric specimens evaluated using the MMT method. Table 9 presents the analysis of variance (ANOVA) for each specimen along with the average moisture transport behavior within the 95% confidence interval. The average values between the three fabric specimens were statistically significant, respectively, as F_0_(V/Ve) > F(2, 12, 0.95) and *p* < 0.05. Figure 9 presents four parameters (wetting time, absorption rate, maximum wetted radius (MWR), and spreading speed) of the three fabric specimens measured using MMT.

The wetting times of KFD PET/Co-PET (specimen 2) and KCC PET/Co-PET (specimen 3) were shorter than that of the KSD PET/Co-PET (specimen 1) (Figure 9a). This suggests that the moisture absorption property of the high wt.% (2.5 wt.%) inorganic particle-embedded fabrics (specimens 2 and 3) is superior to that of the low wt.% (0.36 wt.%) inorganic particle-embedded fabric (specimen 1). In addition, KCC PET/Co-PET exhibited a shorter wetting time than that of KFD PET/Co-PET, meaning that KCC PET/Co-PET has better moisture absorption properties than KFD PET/Co-PET. These results might be explained by the rapid drying characteristics of absorbed moisture in the KFD PET/Co-PET and KCC PET/Co-PET yarns because of the presence of more numerous and larger craters on the constituent fiber surface in the KFD and KCC PET yarns with high hollowness rate (Figure 8 and Table 6), i.e., the rapid drying of absorbed moisture due to the presence of more and larger craters (i.e., high hollowness rate) accelerates the capillary wicking toward multiple (particularly horizontal and vertical) directions in the fabrics, resulting in a shorter wetting time. The present result is consistent with the previous findings [37], where an Al_2_O_3_-embedded hollow PET fabric exhibited superior moisture management performance, as measured by MMT, compared to those containing ZnO, ZrC, and ATO inorganic particles. These results suggest that the CaCO_3_-embedded hollow yarns with a hollowness rate of approximately 30% developed in this study could be commercially applicable to lightweight sportswear.

On the other hand, the absorption rates of specimens 2 and 3 were greater than that of specimen 1 (Figure 9b), which was attributed to the faster-drying property of the KFD PET/Co-PET and KCC PET/Co-PET fabrics than that of the KSD PET/Co-PET fabric, as shown in Figure 9a. In addition, the absorption rate of the KFD PET/Co-PET fabric was less than that of the KCC PET/Co-PET fabric, indicating that the moisture absorption of the KFD PET/Co-PET fabric was inferior to that of the KCC PET/Co-PET fabric. This was attributed to the smaller craters (i.e., low hollowness rate) due to the smaller particle size (0.1–0.3 μm) of TiO_2_ embedded in the KFD PET/Co-PET yarns than that (0.8 μm) of CaCO_3_ embedded in the KCC PET/Co-PET yarns, which was similar to the explanation presented previously for the wetting time. Figure 9c shows the MWR of the three fabric specimens, which is related to the drying property and affects the drying time of the fabric [46]. The MWR of the KFD PET/Co-PET and KCC PET/Co-PET fabrics was greater than that of the KSD PET/Co-PET fabric. Moreover, the KFD PET/Co-PET fabric exhibited a shorter MWR than the KCC PET/Co-PET fabric. Hence, the drying properties of the KFD and KCC PET/Co-PET fabrics were superior to that of the KSD PET/Co-PET fabric, whereas the drying property of the KFD PET/Co-PET fabric was inferior to that of the KCC PET/Co-PET fabric. These results showed that the moisture dropped in the MMT apparatus was absorbed and dried rapidly because of the presence of more and larger craters on the KFD and KCC/Co-PET yarn surfaces than the KSD PET/Co-PET yarn surface, which then penetrated rapidly into the fabrics, resulting in a short wetting time and an increase in the absorption rate and MWR.

The spreading speed (mm/s) of the KFD PET/Co-PET and KCC PET/Co-PET fabrics was faster than that of the KSD PET/Co-PET fabric, as shown in Figure 9d. This result was attributed to the superior drying characteristics because of the presence of more and larger craters on the KFD and KCC/Co-PET yarn surfaces than the KSD PET/Co-PET yarn surface, in which the absorbed moisture penetrated rapidly into fabrics, resulting in faster spreading. In addition, the KCC PET/Co-PET fabric exhibited faster spreading than the KFD PET/Co-PET fabric for the above-mentioned reason. By contrast, KCC PET/Co-PET fabric had numerous cracks on the yarn surfaces, as shown in Figure 7, and exhibited many eluted fragments and broken pieces after elution because of the larger particle size of CaCO_3_, raising some concerns regarding durability while wearing a garment. Therefore, the KFD PET/Co-PET fabric has potential commercial applications in high-performance sportswear with good moisture absorption and rapid drying.

### 3.4. Heat Retention Rate (I) of the Three Fabric Specimens

Understanding how the particle size and amount embedded in the sheath/3-core yarns and the hollowness rate of the fabrics affect the heat retention rate of the hollow yarn fabric is essential to evaluating the thermal wear comfort of the high-performance fabric for sportswear clothing. The heat retention rate was measured using the KES-F7 (Kato Tech Co., Ltd., Kyoto, Japan) apparatus and compared with the particle size and amount embedded in the sheath/3-core hybrid yarns and the hollowness rate of the fabric specimens. Table 10 lists the heat retention rate of the three fabric specimens. ANOVA was conducted to verify the statistical significance using an F-test, as shown in Table 10. An F-test was performed on the three fabric specimens for the average value with a 95% confidence interval. The average heat retention rate of the three fabric specimens was significant because F_0_ (V/V_e_) > F(2, 12, 0.95) and *p* < 0.05.

**Figure 10:**
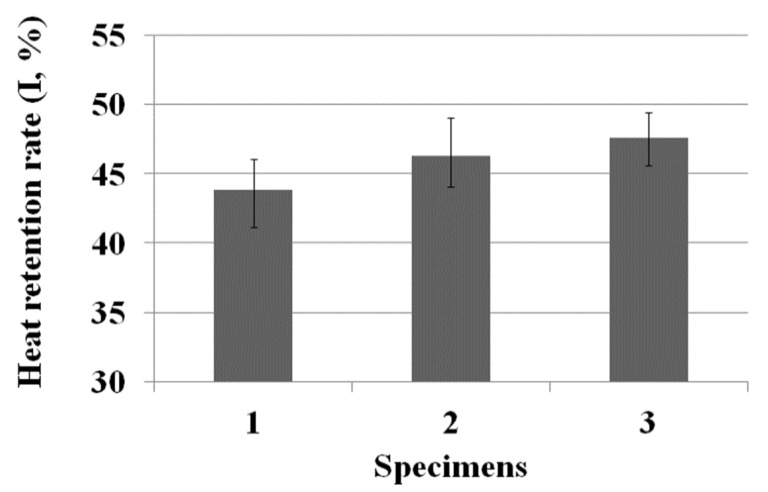
Heat retention rate (I) of the three fabric specimens.

Figure 10 shows the heat retention rate (I) of the three fabric specimens. The I values of the KFD PET/Co-PET and KCC PET/Co-PET fabrics (specimens 2 and 3) were higher than that of the KSD PET/Co-PET fabric (specimen 1), which was attributed to the higher hollowness rate (Table 6) of the KFD and KCC PET/Co-PET fabrics compared to the KSD PET/Co-PET fabric. Hence, the wide space in the yarns with a high hollowness rate entraps more air and prevents heat flow from the inner to the outer layer of the fabrics, resulting in a higher heat retention rate of the high hollow fabric specimens 2 and 3. A previous study [37] reported that an Al_2_O_3_/ATO-embedded hollow fabric exhibited a heat retention rate (I) of 38.60. The significantly higher value (47.62) observed in the present study suggests that the three-hole hollow structure and the incorporation of CaCO_3_ inorganic particles contributed effectively to the superior heat-retaining property of the developed yarns and fabrics. In addition, the I value of the KCC PET/Co-PET fabric was higher than that of the KFD PET/Co-PET fabric because of the higher hollowness rate of the KCC PET/Co-PET fabric, and partly because of the high heat shielding effect of the more and greater craters on the surface of the KCC PET/Co-PET yarn. Accordingly, more air is entrapped in the larger and more numerous craters in fabric specimen 3, enabling complex heat flow from the inner to the outer layers of the fabric, resulting in a high heat-shielding effect (i.e., high heat retention rate) of the KCC PET/Co-PET fabric. Nevertheless, as mentioned previously, the KCC PET/Co-PET fabric has many eluted fragments, broken pieces, and cracks on the yarn surface after elution, which can affect wear durability. Therefore, given the market applications for winter sportswear, the KFD PET/Co-PET fabric, with no durability problem and a relatively high heat retention rate, is useful and suitable for lightweight sportswear clothing with a high hollowness rate.

### 3.5. Tactile Hand Feel with the Wearing Performance of the Three Fabric Specimens

**Figure 11:**
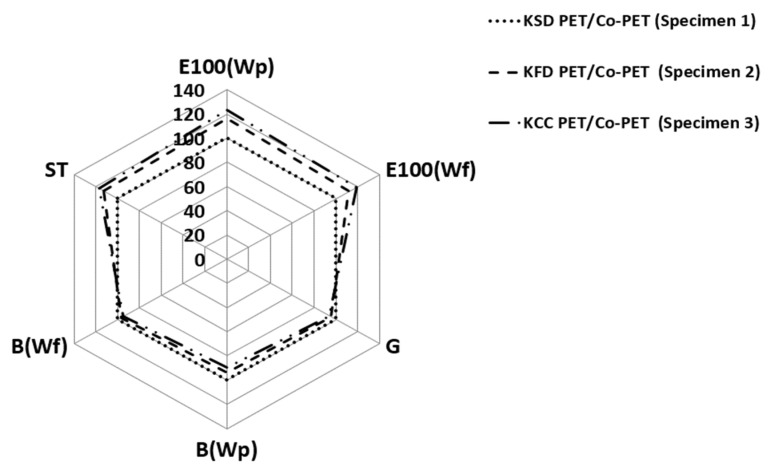
Diagram of the relative mechanical properties of the specimens.

Inorganic particle embedding to impart hollowness to the constituent yarns in the fabrics must not affect their desirable properties, such as tactile hand, wearing performance, and durability for fitness, while wearing the garment as sportswear. This study examined how the particle sizes embedded in the yarns, their embedded amounts, and the hollowness rates of the fabrics affect the tactile hand feel and wearing performance, including durability while wearing clothing, according to the mechanical properties of the fabric measured using a FAST system (CSIRO., Ltd., Eveleigh, Australia). Table 11 lists the mechanical properties of the three fabric specimens. Figure 11 presents the relative mechanical properties of the three fabric specimens. E, B, G, and ST of fabric specimens 2 and 3 were plotted as a ratio to those of fabric specimen 1.

The E values of specimens 2 and 3 were greater than that of specimen 1 (Figure 11). In particular, the G value of these fabrics was less than that of specimen 1, meaning that KFD and KCC PET/Co-PET fabrics are more extensible and less rigid than KSD PET/Co-PET fabrics. This was attributed to the higher hollowness rate of KFD and KCC PET/Co-PET fabrics than that of KSD PET/Co-PET fabric, which might stimulate in-plane deformation under tensile and shear forces, resulting in higher extensibility and lower shear rigidity, i.e., greater extensibility and lower shear rigidity. In addition, this result was assumed to be partly caused by the more (due to the higher wt.%, 2.5 wt.%) and greater (due to the larger particle size, 0.8 μm) craters with cracks on the fiber surface of the KFD and KCC PET/Co-PET yarns, which makes the hollow yarn more flexible, resulting in more extensibility and less rigidity. On the other hand, the E and G values of the KCC PET/Co-PET fabric were higher and lower than those of the KFD PET/Co-PET, respectively, which might be explained by a similar reason to that presented above. The compressibility (ST) of specimens 2 and 3 was higher than that of specimen 1, and specimen 3 showed higher compressibility than specimen 2, i.e., they are more compressible than specimen 1. This could be explained by the longitudinal and compressional deformations of the fabric coinciding with each other in the deformation mechanism, and they might be stimulated by more numerous and larger craters with cracks on the surface of the KCC PET/Co-PET hollow yarns, resulting in higher extensibility and compressibility.

The bending rigidity (B) of specimens 2 and 3 was smaller than that of specimen 1 because of the shorter deflection of the bending length (C in Equation (4)) caused by the higher hollowness rate with less rigidity and lighter weight because of the higher elution rate), which resulted in a smaller B of specimens 2 and 3. The KCC PET/Co-PET fabric (specimen 3) exhibited smaller bending rigidity than the KFD PET/Co-PET (specimen 2) fabric. This was also attributed to the higher hollowness rate because of the larger particle sizes (0.8 μm) of the CaCO_3_ embedded in the KCC PET/Co-PET yarns and partly attributed to cracks on the surface of the KCC PET/Co-PET yarns, which was verified by SEM images of KCC PET/Co-PET yarns (Figure 7).

Considering these mechanical properties, 2.5 wt.% inorganic particles embedded in the sheath of the sheath/3-core yarns (KFD and KCC PET/Co-PET yarns) were assumed to impart a soft tactile hand feel to the hollow fabric compared to the 0.36 wt.% embedded one (KSD PET/Co-PET yarn). Nevertheless, the durability (i.e., wearing performance) while wearing clothing from KCC PET/Co-PET fabric is an issue. On the other hand, the durability (i.e., wear performance) of clothing made from KCC PET/Co-PET fabric remains a concern. Accordingly, further study will be needed to determine the abrasion resistance before and after repeated washing cycles.

## 4. Conclusions

The main objective of the study was to develop inorganic particle-embedded 3-core PET/Co-PET bicomponent yarns and fabrics for use in lightweight sportswear. The results are as follows. The elution characteristics of the three bicomponent yarns (KSD, KFD, and KCC PET/Co-PET) were examined to obtain a high hollowness PET yarn according to the elution conditions, and the optimal spinning and elution conditions were decided. The tenacity and breaking strain of the three sheath/3-core bicomponent yarns produced in this study appeared similar to commercialized regular PET yarns, meaning that the spinnability and weavability of the three bicomponent yarns were relatively good. Hence, there were no issues (no broken filaments or end breakage) during spinning and weaving. The hollowness ratio of the KCC PET/Co-PET fabric was greater than that of the KFD PET/Co-PET and KSD PET/Co-PET fabrics because of the higher wt.% (2.5 wt.%) of the CaCO_3_ particles in the KCC PET/Co-PET yarns than that of the TiO_2_ (0.36 wt.%) in the KSD PET/Co-PET yarns embedded in the sheath of the sheath/3-core bicomponent yarns, and partly due to the larger particle size of CaCO_3_ (0.8 μm) than TiO_2_ (0.1–0.3 μm). On the other hand, KCC PET/Co-PET was assumed to have issues with the washing durability during wear because of the large number of cracks on the yarn surfaces. Regarding the wear comfort characteristics of the three bicomponent yarn fabrics, the MMT test indicated that the KFD PET/Co-PET fabric is suitable for high-performance sportswear with good moisture absorption and fast drying. Considering the market applications in winter sportswear, KFD PET/Co-PET fabric is useful for its relatively high heat retention and durability to washing, and is suitable for lightweight sportswear with a high hollowness rate.

Estimating the wearing performance for fitness with tactile hand feel from mechanical properties using a FAST test, the KFD and KCC PET/Co-PET fabrics were assumed to impart a softer tactile hand feel than the KSD PET/Co-PET fabric. Nevertheless, the wear durability of the KCC PET/Co-PET fabric was assumed to have some issues. Accordingly, further study will be needed to determine the abrasion resistance before and after repeated washing cycles. Finally, the present study was carried out on a pilot-scale spinning system. To advance toward large-scale production, the treatment and recycling of the Co-PET core using NaOH is being considered with small- and medium-sized enterprises, while collaboration with a leading industrial partner (Kolon Materials) is underway to develop melt-spinning nozzle technology.

## Figures and Tables

**Table 1:** Details of sheath/3-core bicomponent yarn specimens.

Yarn Specimen (SDY)	Spinneret		Spinning Temperature (°C)	Spinning Speed (m/min)	Linear Density (d/f)	Remarks
	Type	Sheath:Core				
1. KSD PET/Co PET	sheath/3-core (70:30)	KSD PET: Co-PET	293	3910	75/34	0.36 wt.% TiO_2_
2. KFD PET/Co PET		KFD PET: Co-PET	290	3890	75/34	2.5 wt.% TiO_2_
3. KCC PET/Co PET		KCC PET: Co-PET	290	3880	75/34	2.5 wt.% CaCO_3_

Note: KSD: Kolon semi-dull. KFD: Kolon full-dull. KCC: Kolon calcium carbonate.

**Table 2:** Characteristics of the PET chips used in this study.

PET Chips	Characteristics			Remark
	IV	Tg (°C)	Tm (°C)	(wt.%)
KSD PET in the sheath	0.650	80.7	252	TiO_2_ 0.25
KFD PET in the sheath	0.630	72.1	255.2	TiO_2_ 2.5
KCC PET in the sheath	0.655	77.3	256.2	CaCO_3_ 2.5
Co-PET in the core	0.665	81.3	248	TiO_2_ 0.36

Note: IV: intrinsic viscosity. Tg: glass transition temperature. Tm: melting temperature.

**Table 3:** Specifications of the fabric specimens.

Fabric Specimens	Yarn Used		Fabric Density		Fabric Width (cm)		Weave Pattern
	Warp (d/f)	Weft (d/f)	Warp (ends/cm)	Weft (picks/cm)	Gray	Finished	
1. KSD PET/Co-PET	105/46	150/68	37.6	22.3	152.4	133.1	plain
2. KFD PET-Co-PET	105/46	150/68	37.6	22.3	152.4	133.1	plain
3. KCC PET/Co-PET	105/46	150/68	37.6	22.3	152.4	133.1	plain

Note: KSD: 0.25 wt.% TiO_2_ PET. KFD: 2.5 wt.% TiO_2_ PET. KCC: 2.5 wt.% CaCO_3_ PET.

**Table 4:** Elution conditions of the woven fabric specimens.

Fabric Specimens	Concentration of NaOH (wt.%)	Treatment Time (min)	Treatment Temperature (°C)
1. KSD PET/Co-PET	10	0–60 (10 increments)	90, 100, 110
2. KFD PET/Co-PET	10	0–60 (10 increments)	90, 100, 110
3. KCC PET/Co-PET	10	0–60 (10 increments)	90, 100, 110

**Table 5:** Physical properties of PET/Co-PET bi-component yarns.

Yarn Specimens	Linear Density (d)		Tenacity (gf/d)		Breaking Strain (%)		Unevenness (U%)		Wet Thermal Shrinkage (%)	
	SDY	DTY	SDY	DTY	SDY	DTY	SDY	DTY	SDY	DTY
1. KSD PET/Co-PET	74.5	76.1	3.95	3.81	32.7	30.8	0.60	0.95	10.5	16.8
2. KFD PET/Co-PET	75.3	77.2	3.90	3.72	32.1	28.4	0.99	1.21	10.7	17.6
3. KCC PET/Co-PET	77.4	79.7	3.86	3.70	28.8	26.4	1.19	1.32	8.4	14.9

Note: KSD: Kolon semi-dull. KFD: Kolon full-dull. KCC: Kolon calcium carbonate.

**Table 6:** Hollowness and elution rates of the three hollow fabric specimens.

Number of Observed	Specimen 1			Specimen 2			Specimen 3		
	Diameter (μm)		Hollowness Rate (%)	Diameter (μm)		Hollowness Rate (%)	Diameter (μm)		Hollowness Rate (%)
	Core	Sheath		Core	Sheath		Core	Sheath	
1	4.3	14.6	26.0	4.1	13.8	26.5	4.2	14.3	25.9
2	4.2	14.4	25.5	4.2	13.9	27.4	4.3	14.5	26.4
3	4.1	13.9	26.1	3.9	14.2	22.6	5.2	15.2	35.1
4	4.5	14.5	28.9	4.5	13.8	31.9	4.7	14.8	30.3
5	4.5	14.2	30.1	4.3	13.4	30.9	4.5	15.6	25.0
6	3.9	14.2	22.6	4.6	14.5	30.2	4.2	14.7	24.5
7	3.8	13.9	22.4	4.3	14.2	27.5	5.7	14.5	46.4
8	4.0	14.3	23.5	4.2	14.3	25.9	5.0	14.8	34.2
9	4.1	14.2	25.0	4.0	14.1	24.1	4.2	15.4	22.3
10	4.5	14.7	28.1	4.3	15.1	24.3	4.9	15.2	30.8
Mean	4.2	14.3	25.8	4.2	14.1	27.1	4.7	14.9	30.1
Elution rate (%)	31.6			34.2			36.7		

**Table 7:** ANOVA for hollowness between each specimen.

Hollowness	F-Value (F_0_)	F(2, 27, 0.95)	*p*-Value
Diameter (Core)	6.163	3.354	6.24 × 10^−3^
Diameter (Sheath)	10.538	3.354	4.14 × 10^−4^
Hollowness	22.040	3.354	2.11 × 10^−6^

**Table 8:** Moisture absorption and drying properties of the three fabric specimens.

Specimens	Wetting Time (s)		Absorption Rate (%)		Max. Wetted Radius (mm)		Spreading Speed (mm/s)	
	Top	Bottom	Top	Bottom	Top	Bottom	Top	Bottom
1. KSD PET/ Co-PET	3.872	3.567	34.27	31.72	9.1	8.3	2.016	1.825
2. KFD PET /Co-PET	3.516	3.314	36.35	35.08	10.2	9.1	2.214	2.102
3. KCC PET/ Co-PET	3.417	3.216	37.26	36.25	10.8	9.8	2.275	2.214

**Table 9:** ANOVA for moisture transport behaviors between each specimen.

Absorption and Drying Properties		F-Value (F_0_)	F(2, 12, 0.95)	*p*-Value
Wetting time (s)	top	6.530	3.885	0.012
	bottom	4.111	3.885	0.044
Absorption rate (%)	top	5.508	3.885	0.020
	bottom	14.489	3.885	6.31 × 10^−4^
Max. wetted rad. (mm)	top	16.216	3.885	3.88 × 10^−4^
	bottom	15.712	3.885	4.45 × 10^−4^
Spreading speed (mm/s)	top	5.497	3.885	0.02
	bottom	10.502	3.885	0.002

**Table 10:** Heat retention rate and ANOVA results of the three fabric specimens.

Specimens	Heat Retention Rate (I)	F_0_ Value (F_0_)	F(2, 12, 0.95)	*p*-Value
1. KSD PET/ Co-PET	43.82	10.109	3.885	0.003
2. KFD PET/ Co-PET	46.27			
3. KCC PET/ Co-PET	47.62			

**Table 11:** Mechanical properties of the three fabric specimens.

Specimens	E (%)		G (N/m)	B (μN·m)		ST (mm)
	Wp	Wf		Wp	Wf	
1. KSD PET/Co-PET	0.98	0.74	48.2	51.4	58.2	0.024
2. KFD PET/Co-PET	1.14	0.83	46.1	48.3	56.4	0.027
3. KCC PET/Co-PET	1.21	0.88	45.4	46.2	55.2	0.028

## Data Availability

The original contributions presented in this study are included in the article. Further inquiries can be directed to the corresponding author.
